# Ureteroinguinal hernia: an added advantage for laparoscopy in the management of inguinal hernia—a case report

**DOI:** 10.3389/fsurg.2024.1415875

**Published:** 2024-11-06

**Authors:** Mostafa Zain, Ossama Kasem, Mohamed Gamal, Ahmed Tayel, Mohamed Abouheba

**Affiliations:** Department of Pediatric Surgery, Faculty of Medicine, Alexandria University, Alexandria, Egypt

**Keywords:** ureteroinguinal hernia, laparoscopy, hernia (inguinal), hydroureter, extraperitoneal hernia

## Abstract

Different abdominal organs can herniate into the inguinal canal, including the small bowel, colon, appendix, ovaries, and, less commonly, the urinary bladder and fallopian tubes. Herniation of the ureter within an inguinal hernia is a very rare occurrence. To the best of our knowledge, less than 150 cases have been reported in the literature, including only 15 pediatric cases. A 3-month-old boy presented to our clinic with a left inguinal swelling. Ultrasound of the abdomen and pelvis showed grade 4 left hydronephrosis with a dilated tortuous ureter passing through the left inguinal canal. Further investigation revealed that the patient had a left primary obstructing megaureter with a ureteroinguinal hernia. The case was managed with laparoscopic repair of the inguinal hernia and urethrostomy. The current case proves an additional advantage for laparoscopy as it allows proper visualization of the anatomy and identification of atypical hernias, such as ureteroinguinal hernia.

## Background

Inguinal hernia remains the most common surgical problem encountered by pediatric surgeons. The incidence of inguinal hernia in children ranges from 0.8%to 4.4% (1). It represents more than 15% of the workload in contemporary pediatric surgical practice and is generally considered a well-tolerated procedure with a low risk of significant complications (2). Different abdominal organs can herniate into the inguinal canal, including the small bowel, colon, appendix, ovaries, and, less commonly, the urinary bladder and fallopian tubes. Herniation of the ureter within an inguinal hernia is a very rare occurrence. To the best of our knowledge, less than 150 cases have been reported in the literature, including only 15 pediatric cases (3–6).

Since the 1990s, laparoscopic repair of inguinal hernia has gained popularity and has become the standard of care in many centers; however, the traditional open surgical approach is still the most commonly used technique worldwide (7). The use of laparoscopy offers many benefits, such as better cosmetic results, less postoperative pain, faster recovery, shorter hospital stays, and visualization of the contralateral deep inguinal ring. However, due to its increased cost, prolonged learning curve, and longer operative time, it has not achieved the global acceptance seen with other procedures, such as appendectomy and cholecystectomy (7). In this report, we describe the laparoscopic treatment of a ureteroinguinal hernia in a 3-month-old infant, which can be considered another advantage of using laparoscopy for inguinal hernia repair. This manuscript was prepared following the CARE guidelines (https://www.care-statement.org).

## Case presentation

**Figure 1:**
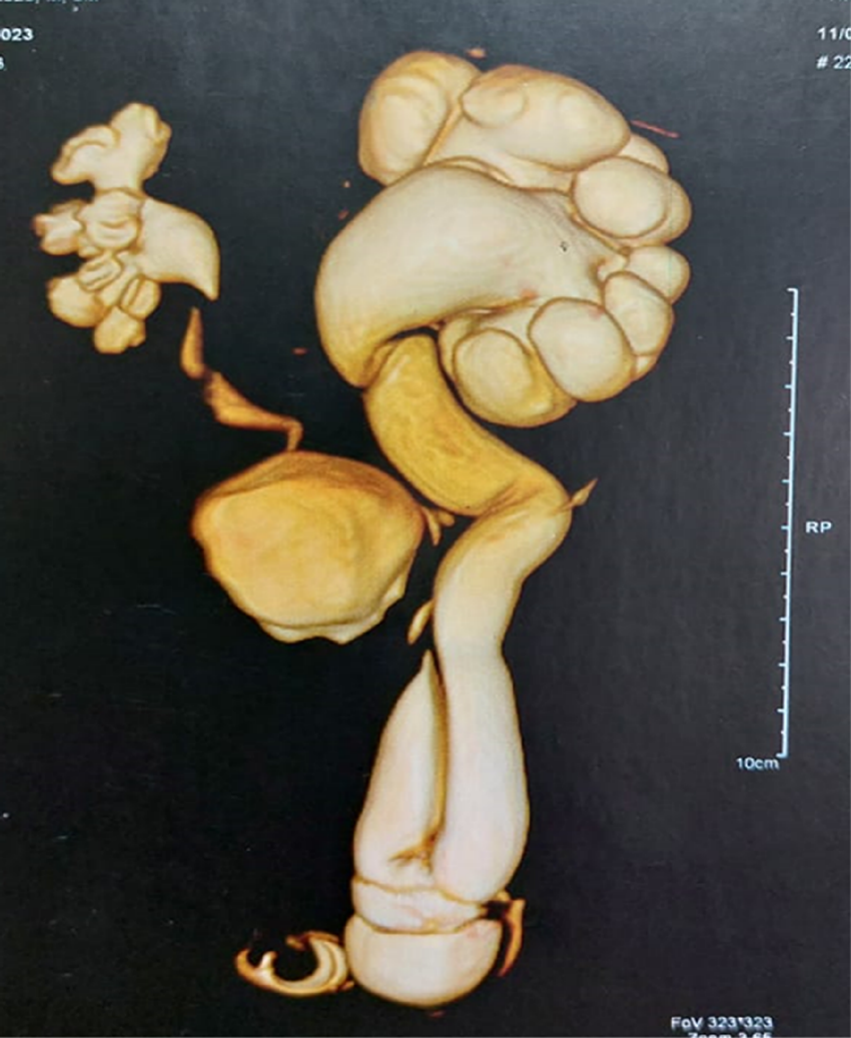
Preoperative magnetic resonance urography showing left severe hydroureteronephrosis with dilated scattered calyces and multiple kinks in the ureter. The dilated ureter extends into a moderately sized, fat-containing inguinal hernia.

Our patient was a 3-month-old boy who was brought to our clinic by his mother who complained that her child had inguinal swelling on the left side. The mother said that she had noticed this swelling for 2 days. The boy was delivered by cesarean section at full term with a birth weight of 3.2 kg. Antenatal ultrasound (US) examinations were unremarkable. The patient's medical, surgical, and family histories were unremarkable. On clinical examination, the boy looked healthy with an irreducible left inguinal swelling. However, there were no signs or symptoms of intestinal obstruction. The provisional diagnosis was thought to be an irreducible left inguinal hernia. US of the abdomen and pelvis revealed a grade 4 hydronephrosis on the left side with a dilated tortuous ureter passing through the left inguinal canal. For better anatomic visualization, magnetic resonance urography (MRU) was carried out. It showed the enlarged left kidney in a normal position, with marked hydroureteronephrosis, dilated scattered calyces, and multiple kinks in the ureter. The dilated ureter extended into a moderately sized, fat-containing inguinal hernia (Figure 1).

To determine whether the ureteric dilatation was due to obstruction or reflux, we performed a voiding cystourethrogram (VCUG), which showed normal bladder filling and voiding without any reflux. Therefore, the diagnosis of left primary obstructing megaureter with ureteroinguinal hernia was made. An isotopic dimercaptosuccinic acid (DMSA) renal scan showed normal homogeneous radioactive uptake in the right kidney, while the left kidney was enlarged in size showing moderate radioactive accumulation. There was no evidence of infection or scarring on either side. The relative uptake was 74% for the right kidney and 26% for the left kidney. The treatment plan included diagnostic cystoscopy, laparoscopic repair of the inguinal hernia, and urethrostomy.

**Figure 2:**
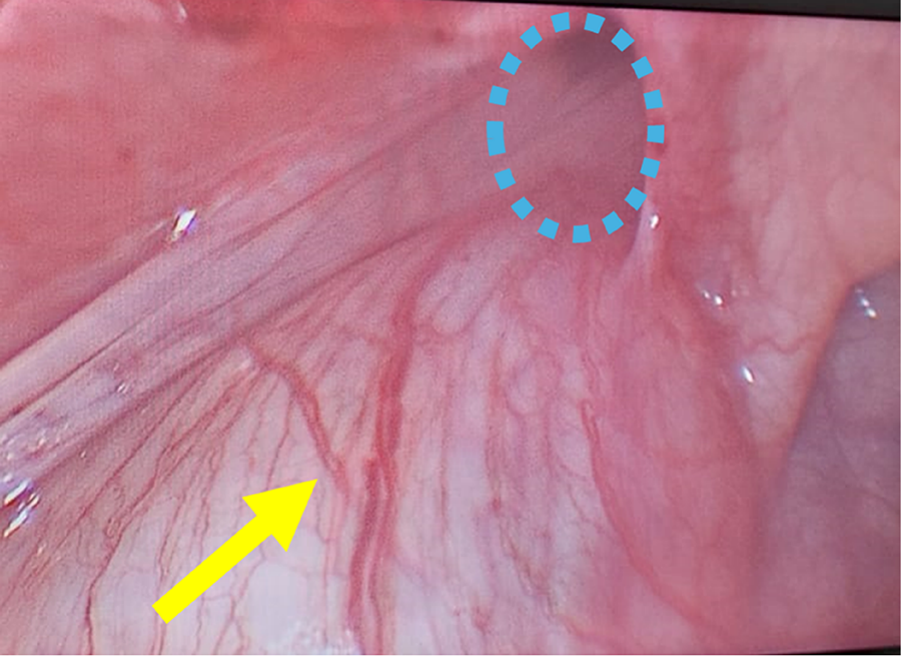
Intraoperative laparoscopic photograph showing a massively dilated and tortuous left ureter (yellow arrow) forming a loop sliding into the left inguinal canal with a small hernial sac (blue dashed circle).

The basic preoperative evaluation, including routine laboratory investigations and cardiac and anesthesia reviews. was normal. Diagnostic cystoscopy showed that the right ureteric orifice was normal and orthotopic, while the left ureteric orifice could not be visualized. After inserting a 5 mm umbilical trocar and introducing a 30° endoscope through the umbilicus, two 3-mm instruments were inserted directly (without trocars) at the right and left mid-clavicular lines, aligned at the level of the umbilicus, to maintain a triangular orientation. Laparoscopic exploration revealed a massively dilated and tortuous left ureter forming a loop sliding into the left inguinal canal with a small hernial sac (Figure 2).

**Figure 3:**
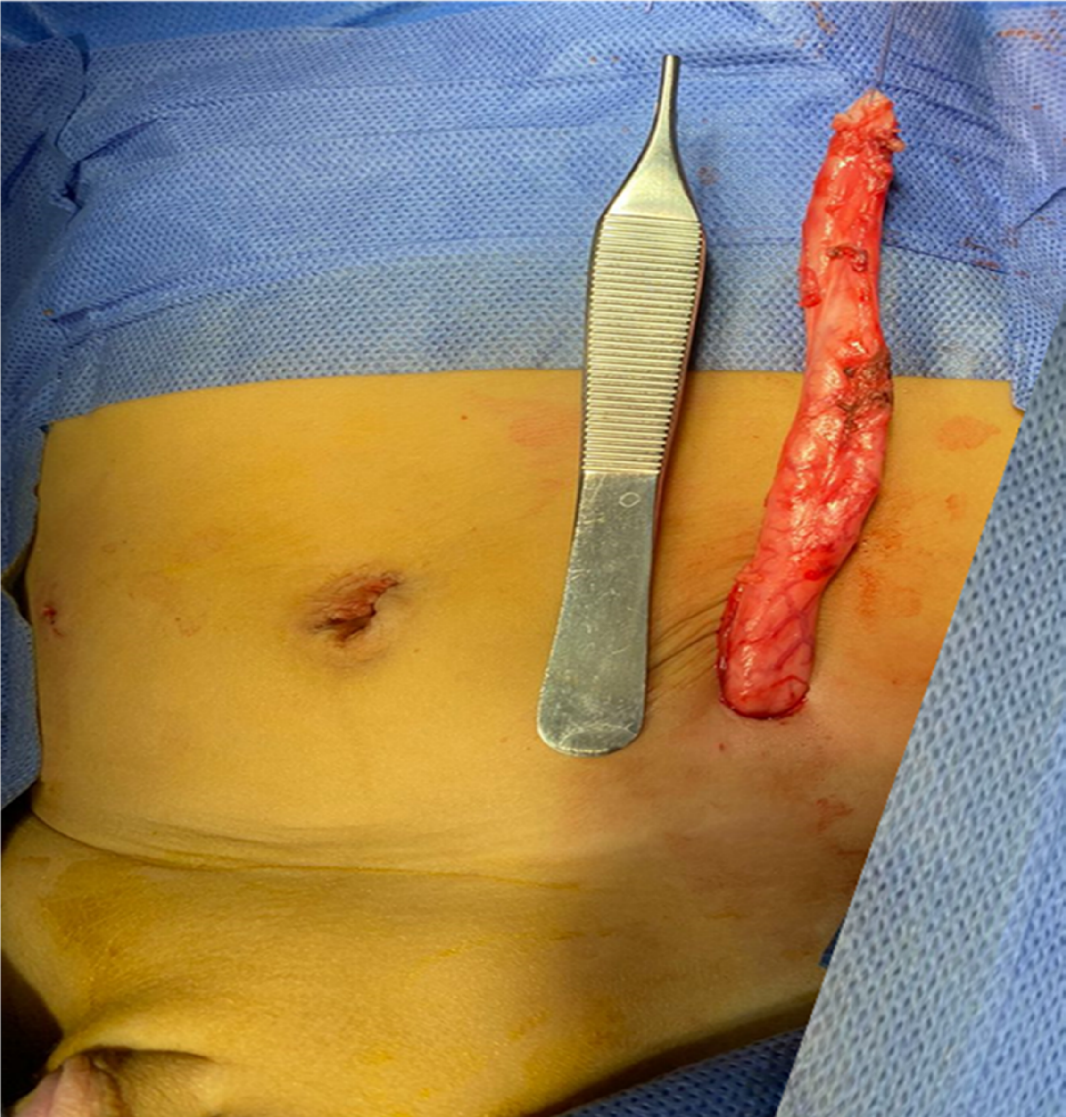
Intraoperative photograph after exteriorization of the ureter through a small incision in the left iliac region.

After disconnection of the hernial sac from the abdominal peritoneum, the ureter was reduced, and the internal inguinal ring was tightened with a single 4/0 prolene suture. The ureter was dissected down to its entrance into the urinary bladder, where it was separated. It was then exteriorized through a small incision in the left iliac region (Figure 3). A ureterostomy was performed after the excision of the excess dilated ureter. The postoperative course was uneventful. The boy resumed oral feeding on the first postoperative day and was discharged on the second day. The plan was to perform extravesical ureteric reimplantation when he reached the age of 1 year.

## Discussion

Inguinal hernia is one of the most common surgical problems in children (8). It is estimated that more than 20 million hernia repairs are performed every year worldwide (9). The major complication of inguinal hernia, if left untreated, is incarceration, which can lead to intestinal gangrene and gonadal atrophy (10). The risk of incarceration in children ranges from 3% to–16% in different studies; however, in premature infants, it is estimated to be as high as 30% (11).

The usual contents of the inguinal hernia include the small intestine, colon, omentum, and ovaries. Less commonly, the appendix (Amyand's hernia) or Meckel's diverticulum (Littre hernia) may be present. In addition, the urinary bladder may herniate into a direct sac or form part of the wall in a sliding hernia (12). These uncommon findings pose a technical challenge, increasing the risk of inadvertent injury to these organs.

Herniation of the ureter into the inguinal canal is a very rare finding (4, 13). It was first reported in 1880 by Leroux as an autopsy finding (14). In 1892, Reichel reported the first case of ureteral hernia discovered during hernia repair (15). In 1937, Dourmashkin made the first preoperative diagnosis of a ureteral hernia using intravenous pyelography (16). A recent review of the literature by Laurie revealed that less than 150 cases were reported in the English literature (3). Cianci et al. reviewed pediatric cases and found 12 cases in addition to their own case (4). Later, two more pediatric cases were reported by Delgado-Miguel et al. and Wishahi (5, 7).

Ureteroinguinal hernia is classified based on the presence or absence of a hernial sac, classifying it as paraperitoneal or extraperitoneal, respectively (17). The extraperitoneal type is less common, accounting for only 20% of cases. The ureter develops from an outgrowth (ureteric bud) that originates from the mesonephric duct and also gives rise to the epididymis, vas deferens, seminal vesicles, and the ejaculatory duct. The extraperitoneal type results from an embryological abnormality in which the ureteric bud separates late from the Wolffian duct as it descends to form the epididymis. The ureter, with a significant amount of retroperitoneal fat, herniates into the inguinal canal in the absence of a hernial sac (18, 19).

On the other hand, the paraperitoneal type accounts for 80% of cases. It can be considered a sliding hernia, as the ureter, being a retroperitoneal structure, is part of the wall of the hernia sac rather than an internal component of the hernia. This type is more commonly associated with a dilated ureter, as seen in cases of vesicoureteral reflux, posterior urethral valve, and obstructing megaureter. It has been suggested that this type may develop due to traction on the ureter caused by abnormally adherent posterior parietal peritoneum (18–20). This was the case with our patient.

Risk factors for ureteroinguinal hernia in adults include obesity, anterior displacement of the ureter from the psoas muscle, scarring from previous hernia repair, and renal transplantation (12). However, the risk factors in the pediatric population are still uncertain. In their review, Cianci et al. suggested congenital pathogenesis in children, noting that the majority of cases had an almost constant association with other congenital anomalies of the urogenital system. They stated that it is difficult to determine whether the dilated ureter found in the majority of cases results from the ureteroinguinal hernia or whether it serves as a predisposing factor, causing ureteric displacement toward the inguinal canal due to its convoluted course (4). In this context, our case involved a left primary obstructing megaureter with a massively dilated tortuous ureter, which may have contributed to the ureteric herniation into the inguinal canal.

Preoperative diagnosis of ureteric herniation is essential for appropriate surgical decisions and to minimize the risk of ureteric injury during operation; however, the majority of cases are identified intraoperatively (18). In the 15 reported pediatric cases, the ureteric injury occurred in 4 out of 7 cases without preoperative diagnosis during inguinal herniotomy, whereas in the other 8 cases with a preoperative diagnosis, no other operative complications were reported.

Ureteroinguinal hernia may be suspected in the presence of urinary symptoms such as dysuria, urinary frequency, or flank pain; however, most patients are asymptomatic (17). The routine preoperative workup for inguinal hernia does not require any specific imaging, as it is considered a straightforward clinical diagnosis and unusual findings are rare. Some authors advise increasing the index of suspicion for a ureteroinguinal hernia when an inguinal hernia presents in a patient with unexplained hydronephrosis or recurrent urinary tract infections, especially in male patients. In addition, if the traction of the testis on clinical examination of the external genitalia is associated with the appearance of an ipsilateral bulge, further evaluation may be warranted. In such cases, an algorithm proposed by Yahya et al. for adult patients suggests considering additional urological evaluation, including US, VCUG, and renal scintigraphy, while cystoscopy remains optional (12). A computed tomography (CT) scan or MRU may be considered in the pediatric population if US does not lead to a preoperative diagnosis (17). In our case, the diagnosis was suspected by US and confirmed by MRU.

During open surgery for an inguinal hernia, the possible presence of the ureter should be suspected if unusual structures are encountered in the operative field, especially if they are found outside the hernial sac. Consideration should be given to the probability of herniating retroperitoneal structures, especially the ureter if the hernia contains fat that does not fit the criteria of a cord lipoma. In such cases, cautious fat reduction should be carried out. If complete reduction of the fat is not possible, careful dissection to the level of the deep ring is necessary to prevent ureteric damage (12, 20). In this context, the laparoscopic approach is ideal, as it allows for a thorough inspection of the inguinal and pelvic regions (4).

Failure to achieve a preoperative diagnosis and recognize ureteric involvement intraoperatively may result in high morbidity and even mortality. The ureter, buried beneath a thick layer of fat, may be mistaken for extraperitoneal fat or a cord lipoma, resulting in injury from blind clamping and division (21). Given the number of hernia surgeries performed worldwide, routine CT scans, or MRU, are not justified; however, they are recommended for patients with abnormal renal function tests or urinary symptoms. Therefore, the laparoscopic approach offers a great advantage by providing proper visualization of the anatomy before division (21).

Laparoscopic repair of inguinal hernias has gained popularity worldwide over the past three decades; however, it is still less common than the open approach due to its high cost, longer operative time, conversion from an extraperitoneal procedure to an intraperitoneal one, and longer learning curve (22, 23). However, laparoscopy offers many advantages over the open approach, such as detection and repair of contralateral hernias during the same session, magnification, reduced manipulation of the vas deferens and testicular vessels in male patients, less postoperative pain, and a better cosmetic outcome. It is also considered to be the best option for managing recurrent inguinal hernias (22, 24). In addition, it allows for the identification of atypical hernias, such as ureteroinguinal hernias, even in cases without preoperative diagnosis (4, 21).

The management of ureteroinguinal hernias in adults is debatable as the majority of authors recommend hernia repair with retroperitoneal relocation of the ureter, while others propose conservative management (25). On the contrary, surgical repair is essential for pediatric ureteroinguinal hernias, as they are usually paraperitoneal and involve a hernial sac that requires independent repair from the associated ureteral herniation. In addition, it is difficult to discriminate between the paraperitoneal and extraperitoneal types preoperatively (4).

If there is no associated megaureter or other anomalies, the patient will require only hernial repair with the relocation of the ureter to the retroperitoneum (25, 26). Alternatively, in cases involving a severely dilated ureter, management will depend on the primary pathology. Various procedures have been described in the reported pediatric cases, including partial resection, ureterostomy, ureteric reimplantation, and transureteroureterostomy (27–30). In our patient, we performed a temporary ureterostomy and planned for future ureteric reimplantation as a result of the patient’s young age and the significant degree of ureteric dilatation. Reimplantation of a grossly dilated ureter into a small infant urinary bladder could be a challenging operation in young infants, as the discrepancy between the dilated ureter and the small bladder raises concerns about potential iatrogenic bladder dysfunction (31, 32). For infants under 1 year of age with a primary obstructing megaureter that requires intervention, the British Association of Paediatric Urologists’ consensus statement supports temporizing measures such as stenting, balloon dilatation, temporary refluxing reimplantation, or cutaneous ureterostomy. It recommends delaying definitive reimplantation surgery until the child is 1 year old (33).

## Conclusion and recommendations

The current case demonstrates the importance of preoperative evaluation and diagnosis of ureteroinguinal hernia. It also emphasizes how this rare condition may go unnoticed during the standard preoperative evaluation for elective day-case inguinal hernia repair. We recommend performing an US evaluation in cases of irreducible inguinal hernia that do not present with signs of intestinal obstruction.

It also highlights an additional advantage of laparoscopy, as it allows for proper visualization of the anatomy and identification of atypical hernias, such as ureteroinguinal hernias, even in cases without a preoperative diagnosis, as well as the other reported advantages of the laparoscopic approach.

## Data Availability

The original contributions presented in the study are included in the article/Supplementary Material, further inquiries can be directed to the corresponding author.
